# Workaholism on Job Burnout: A Comparison Between American and Chinese Employees

**DOI:** 10.3389/fpsyg.2018.02546

**Published:** 2018-12-11

**Authors:** Francis Cheung, Catherine S. K. Tang, Matthew Sheng Mian Lim, Jie Min Koh

**Affiliations:** ^1^Department of Applied Psychology, Lingnan University, Tuen Mun, China; ^2^Department of Psychology, National University of Singapore, Singapore, Singapore

**Keywords:** workaholism, job burnout, cross-cultural differences, Chinese, American, burnout, moderation

## Abstract

Past research frequently reports significant relation between workaholism and job burnout, and some studies further indicate workaholism varies across countries. Surprisingly, there is no study that directly examines whether country moderates the workaholism-burnout association. To address this research question, we have collected independent work samples from two culturally diverse countries, namely the People’s Republic of China and the United States. A total of 2243 participants (1243 American respondents and 1000 Chinese respondents) were recruited. Preliminary group comparison suggested that there were statistical differences among participants from different industries on the key variables, including workaholism, job demands, autonomy and emotional exhaustion. Therefore, we have divided our participants into three subsamples [i.e., (1) natural resources, mining and construction industry, (2) manufacturing industry, and (3) service industry] and separate analyses were conducted. In the moderated regression analyses, workaholism significantly predicted two dimensions of job burnout, namely emotional exhaustion and depersonalization, even when job demand and job autonomy were controlled. Finally, although two significant moderating effects were found, there was a lack of consistent empirical support to the hypothesized moderating effect of country on workaholism-burnout association. Implications and limitations were discussed.

## Introduction

Work is a central component in adults’ life: It satisfies the fundamental needs of humans, including survival, relatedness to others, and self-determination (Blustein, 2008). Without work, such as when individuals experience job loss, people would often struggle with mental health problems (e.g., Vinokur et al., 2000; Lucas et al., 2004). Some individuals devote excessive hours in their work to an extent that is beyond what is expected. These individuals are often regarded as “workaholics.” Studies on workaholism and its potential impact is accumulating (e.g., Ng et al., 2007; Bakker et al., 2013; Matsudaira et al., 2013; Andreassen et al., 2016; Clark et al., 2016). However, there is a paucity of studies exploring whether workaholism relates to psychological well-being differently across countries (e.g., Harpaz et al., 2002). Guided by the conservation of resource model (Hobfoll, 1989), this study examines the association between workaholism and job burnout. Besides, we will explore whether country of origin moderates the effect of workaholism on burnout. We have collected work samples from China and the United States to compare the role of workaholism in predicting job burnout in these two culturally different work groups.

### Defining Workaholism

Since the introduction by Oates (1971), workaholism has been discussed in the academia and other arena for decades. To be classified as a “workaholic”, individual should has strong obsessions or need for work which has become so excessive that it creates disturbance with his or her personal health and happiness, interpersonal relations, and social functioning (Oates, 1971). Although it is clear the majority of researchers conceptualize workaholism as an addiction to work (Clark et al., 2016), there is still a lack of consensus on its definition and the true nature of workaholism (McMillan and O’Driscoll, 2006). For example, some researchers suggested concrete number of hours as the cutoff to differentiate employees with workaholism (e.g., Mosier, 1983), other studies defined workaholism by considering an employee’s attitude toward work, such as inclination to invest additional time and energy on work beyond the required limit (e.g., Spence and Robbins, 1992; Mudrack, 2004; Ng et al., 2007). Moreover, there are still debates on whether the construct should be treated as a uni-dimensional (e.g., Andreassen et al., 2010) or multi-faceted variable (e.g., Spence and Robbins, 1992; Schaufeli et al., 2009b; Taris et al., 2010). Finally, there is still an ongoing discussion on whether there are “positive” and “negative” workaholism (e.g., Naughton, 1987; Scott et al., 1997). To further complicate the understanding of workaholism, researchers suggest that it shared a lot of features with other vocational constructs, such as work engagement (Schaufeli et al., 2002, 2006, Schaufeli et al., 2002) and work centrality (e.g., Bal and Kooij, 2011). For example, engaged workers have a strong sense of energetic and effective connection with their work activities and they see themselves as able to deal well with the job demands (Taris et al., 2010). Because engaged employees work very hard and feel engrossed in their job, they would usually invest much more time and effort in their work. The heavy investment in their work duties thus resembles with workaholic behaviors.

While long work hours can be a result of intrinsic/personal factors (e.g., workaholic, high work engagement), equally important is the effect of extrinsic factors, such as organizational culture or work role changes. An example is the prolonged work hours resulting from work intensification. Work intensification refers to the process of raising the expected workload of an employee by increasing the number of tasks to be accomplished or through shortening the time allowed to finish the assigned work tasks. With more roles and tasks assigned, it is inevitable that employees have to spend longer work hours to complete all the assigned job duties. Longer work hours will lead to higher fatigue, more work stress and work life imbalance, which will in turn endanger employees’ occupational well-being (Macky and Boxall, 2008; Boxall and Macky, 2014).

To sum up, long work hours or heavy work investment can be a result of intrinsic factors (e.g., workaholic) and/or extrinsic factors (e.g., work intensification). Snir and Harpaz (2012) proposed a heavy work investment model to delineate situational-driven high work investment (e.g., work intensification, financially driven heavy work investment) and dispositional-driven high work investment (e.g., workaholism). Under this framework, workaholics can be conceptualized as a subtype of heavy investment workers in which they are motivated primarily by internal urge instead of situational factors. When compared to other heavy work investors, workaholics also differ in terms of the level of autonomy on their excessive work involvement. For example, engaged workers choose to devote more work hours because they find the job meaningful and fun. However, workaholic do not have much autonomy over the compulsion to work and they usually do not enjoy the work process (Snir and Harpaz, 2012).

In this study, the focus will be limited to workaholism, which is defined as “being overly concerned about work, to be driven by strong and uncontrollable work motivation, and to spend so much energy and effort into work that it impairs private relationships, spare-time activities and/or health” (Andreassen et al., 2010, p. 8). Our goal is to examine whether workaholism relates to burnout, and whether such association varies across different cultural contexts.

### Workaholism and Burnout

Although some researchers suggested that there is a positive side of workaholism (e.g., Hu et al., 2014), most studies focus on the detrimental effect. A common occupational health outcome relates to workaholism is job burnout. (e.g., Montgomery et al., 2003; Schaufeli et al., 2008, 2009a). Job burnout is a prolonged response to chronic emotional and interpersonal stressors on the job and is defined by three dimensions of emotional exhaustion, depersonalization, and lack of personal accomplishment (Maslach et al., 2001). Emotional exhaustion refers to feelings of being overextended and depleted of emotional and physical resources, depersonalization reflects negative and detached responses, and sense of personal accomplishment represents feelings of competence and achievement at work. The significant relation between workaholism and burnout has been reported in a recent meta-analysis by Clark et al. (2016). In particular, meta-analytic results showed that there was a stronger correlation between workaholism and emotional exhaustion (ρ = 0.42) and depersonalization (ρ = 0.29), while the correlation on lack of personal accomplishment was substantially lower (ρ = -0.03).

In the present study, we adopted the Conservation of Resources model (COR model, Hobfoll, 1989) as the framework to hypothesize the association between workaholism and job burnout. According to the COR, stress occurs when individuals experience threatened or actual loss of valuable resources. Individuals will then strive to minimize resource loss as well as to retain, protect, and foster valued resources. Failure to retain and replenish resources drain will lead to psychological strain. Compared to non-workaholic, workaholics are driven to work because of strong work compulsion (Graves et al., 2012). They are also constantly thinking about their work even when they are not at work (Scott et al., 1997; Clark et al., 2016). Such compulsion of work and the inability to disengage themselves from work will inevitably create more stress for workaholics. The excessive work hours have also reduced workaholics’ opportunity to engage in active coping behaviors to replenish lost energetic resources, such as taking breaks for recovery (Ng et al., 2007; Bakker et al., 2013). Taken together, workaholics have to constantly invest their resource (i.e., cognitive, emotional, physical) in work but they fail to replenish their resources via resource restoration activities. They will thus result in more job burnout when compared to non-workaholics.

Hypothesis 1: Workaholism will be positively related to job burnout.

### Workaholism on Job Burnout Across Countries

Although workaholism is hypothesized to correlate with job burnout, such association is determined by multiple factors, such as gender (Eagly, 1987; Pleck, 1993), personality (e.g., Burke et al., 2006; Andreassen et al., 2010), work characteristics (e.g., Molino et al., 2016), among others. As suggested by the COR model, when we want to understand the stress-coping process, it must be understood within the frame of individuals in social context (e.g., family, neighborhood, organization). In other words, the country that the employees belong to or the boarder cultural context will inevitably influence how individuals cope with stress which would lead to different outcomes.

Indeed, there have been several empirical studies to explore the cultural effect on workaholism. Hu et al. (2014) examined workaholism and work engagement across various culturally different countries, including the Netherlands, Spain, Finland, China, and Japan. Their results showed that employees in the Western cultures tend to be more work engaged than employees in the Eastern cultures. In terms of workaholism, employees in China tend score higher than employees in Western European countries. Snir and Harpaz (2006) examined workaholism in Belgium, Israel, Japan, the United States, and the Netherlands. Defining workaholism by the total work hours and perception of work centrality, Japanese employees reported the highest work hours in the four studied countries, and they were also reporting the highest level of work centrality. Finally, Schaufeli et al. (2009a) examined workaholism in the Netherlands and Japan. Defining workaholism as the combination of working excessively and working compulsively, these authors found that workaholism was significantly related to burnout. However, the correlation between workaholism and work engagement was more salient in the Dutch sample but not in the Japanese sample. Although these studies document the workaholism phenomenon in different cultural contexts, the focus and objective differ widely: Some of these studies focus on the measurement invariance across countries (e.g., Schaufeli et al., 2009b), other studies are primarily interested to explore the magnitude or prevalence of workaholism across different cultures (e.g., Snir and Harpaz, 2006; Hu et al., 2014). Surprisingly, there is no empirical study that directly examine whether the country of origin moderates the workaholism- job burnout association. In particular, is the association between workaholism and burnout more prominent in some countries whereas in some countries, these associations are weakened?

To address this knowledge gap, we will examine whether the association between workaholism and job burnout differs across two culturally different countries, namely the United States and China, which are prime examples of individualistic and collectivistic cultures, respectively (Hofstede, 1980). When facing work stress, resources available would influence how the individual perceive the work stress experience and the subsequent coping strategy (Aldwin, 2007). People in individualistic culture tend to respond and cope with the problem directly and on their own when they are dealing with stress (e.g., Weisz et al., 1984; Tata and Leong, 1994). For people in collectivistic culture which emphasizes on the connectedness with significant others, these individuals would often seek support from members in the social network (Tata and Leong, 1994). Based on the COR model, the availability of social support or social capital will be important resources to offset the detrimental effect of work stressor. Workaholic employees in Chinese culture may have more resources (e.g., familial support and social support from friends) than their American counterparts to offset the resource drain.

Taken together, the different interpretation of workaholism and the availability of social capital may affect the job burnout experience in these two culturally different background. It is hypothesized that under workaholism, employees in collectivistic culture (Chinese employees) will report lower burnout when compared to American employees (individualistic culture).

Hypothesis 2: The association between workaholism and job burnout will be moderated by the country. In particular, the effect of workaholism on job burnout will be stronger in the United States sample than in the Chinese work sample.

### Control Variables: Job Demand and Job Autonomy

An issue in earlier workaholism studies is the overlook of confounding factors that may affect the workaholism-job burnout association. For example, in the job demand-control model (Karasek, 1979), job burnout is significantly related to job demands and job autonomy (e.g., Demerouti et al., 2001; Crawford et al., 2010; Nahrgang et al., 2011). The same set of factors are also significant correlates of workaholism (e.g., Molino et al., 2016; Andreassen et al., 2017). Therefore, it is highly plausible that the workaholism-job burnout association is influenced by the presence of exogenous variables (e.g., job demand, job autonomy). Thus, in order to tease out the effect of workaholism on job burnout, the perception of job demand, the availability of job autonomy need to be taken into consideration.

## Materials and Methods

The participants were recruited via online platforms. Two crowdsourcing companies, one in the United States (Amazon Mechanical Turk^1^) and one in the PRC (Rakuten AIP^2^) sent emails to invite residents to participate in the current research. Hyperlinks to the web survey were sent to those who consented to participate. Confidentiality was assured and the informed consent of the participants was obtained by virtue of survey completion. Eligible participants were at least 25 years old and were holding full-time jobs at the time of the research. Upon the completion of the online survey, the participants were given a code number to claim their participation fees. A total of 2233 participants joined the current research. The United States sample comprised 1233 participants (671 males and 562 females) with an average age of 37.287 (*SD* = 9.16). Meanwhile, the PRC Chinese sample consisted of 1000 participants (495 males and 505 females) with an average age of 39.93 (*SD* = 8.95). Among these participants, majority were salaried employees (92.8%). The mean age of Chinese participants (*M* = 39.93, *SD* = 8.85) was higher than the American participants (*M* = 38.06, *SD* = 28.89). In terms of industries, 21.3% were working in other services except public administration, 18.2% were employed in manufacturing industry, 17.1% were employed in education and health services, 15.9% were employed in professional and business sector, 11.8% were employed in information technology. Remaining participants were employed in trade, transportation, and utilities (8.5%), natural resources, mining and construction (3.9%), and leisure and hospitality (3.4%). Table 1 presents the details of the demographic information. Group comparison based on industry was conducted to examine if major variable (autonomy, job demands, workaholism and burnout) varied across these industries. ANOVA results suggested that level of job demand (*F* = 10.09, *df* = 7, 2225, *p* < 0.01), autonomy (*F* = 2.26, *df* = 7, 2225, *p* = 0.03), workaholism (*F* = 15.34, *df* = 7, 2225, *p* < 0.01) and emotional exhaustion (*F* = 2.26, *df* = 7, 2225, *p* = 0.03) were significantly different. Due to the heterogeneity of the sample, combining all the participants into an overall sample may lead to biased results. However, running independent analyses based on the eight industries will inflate the Type 1 error and statistical power will be reduced. Therefore, in the subsequent analyses, the eight occupations were re-organized into three main industries, namely natural resources/mining and construction (*n* = 86), manufacturing industry (*n* = 407) and service industry (*n* = 1740). These three industries generally corresponded to the classic three-sector economic production model.

**Table 1:** Analysis with type of industry.

			Job			Emotional		Personal
	*N*	%	demand	Autonomy	Workaholism	exhaustion	Depersonalization	Accomplishment
Natural Resources and Mining/Construction	86	3.9	2.64	3.06	20.03	11.95	11.81	9.02
Manufacturing	407	18.2	2.86	2.96	21.87	12.52	13.74	9.97
Trade, Transportation, and Utilities	189	8.5	2.72	2.96	19.90	12.36	13.14	9.68
Information Technology	263	11.8	2.53	2.89	17.90	13.29	13.31	8.70
Professional and Business Services/Financial Activities	355	15.9	2.54	2.95	17.66	12.63	13.12	8.80
Education and Health Services	381	17.1	2.67	2.87	19.39	13.71	13.29	8.95
Leisure and Hospitality	76	3.4	2.70	2.66	17.88	15.76	15.92	8.72
Other Services (except Public Administration)	476	21.3	2.62	2.90	19.07	13.22	13.65	9.59
Total	2233	100	2.66	2.92	19.34	13.06	13.44	9.28

### Measures

#### Job Demands

Job demands were measured by items adapted from the job content questionnaire (Karasek et al., 1998). The scale consists of four items and it was used to assess an individual’s perception of work load and overall job demand. A sample item included “your job requires you to work very fast.” Higher score indicates higher perceived job demands. The alpha coefficient of this scale in the American and Chinese samples were 0.66 and 0.71, respectively.

#### Job Autonomy

Job autonomy was measured by items adapted from the job content questionnaire (Karasek et al., 1998). The scale consists of two items and it was used to assess an individual’s perception of their level of job autonomy over the job duty. A sample item included “It is possible for you to decide for yourself what should be done in your work.” Higher score indicates more autonomy and control over the job responsibility. The alpha coefficient of this scale in the American and Chinese samples were 0.73 and 0.82, respectively.

#### Workaholism

Workaholism was measured by the work addiction scale. The scale consists of seven items and it was used to assess an individual’s perception of excessive work behaviors. Sample items included “Spent much more time working than initially intended” and “become stressed if you have been prohibited from working.” Higher score indicates higher workaholism inclination. The alpha coefficients of this scale in the American and Chinese samples were 0.86 and 0.84, respectively.

#### Job Burnout

The 16-item Maslach Burnout Inventory-General Survey (MBI-GS) was used to assess employee burnout. The instrument consists of three subscales that measure emotional exhaustion, depersonalization, and personal accomplishment. Sample item of the above three dimensions were “you feel emotionally drained from my work” (emotional exhaustion), “you have become more cynical about whether your work contribute anything” (depersonalization), and “I feel I’m not positively influencing other people’s lives through my work” (personal accomplishment, reversed scored). Participants rated their feelings and attitudes toward their work on a 4-point scale, ranging from 1 “Never” to 4 “Always.” Higher scores indicate higher levels burnout. The alpha coefficients were 0.95, 0.90, and 0.83 for emotional exhaustion, depersonalization and lack of personal accomplishment for the Chinese sample, respectively. For the United States sample, the alpha coefficients were 0.95, 0.82, and 0.88 for emotional exhaustion, depersonalization and lack of personal accomplishment, respectively.

### Descriptive Statistics

Participants were asked to indicate their age, gender, occupation, and education.

## Results

### Assessment of Common Method Variance

All data in the present study were collected via self-administered questionnaires. Therefore, common method variance may inflate the strength of observed relationships (Podsakoff et al., 2003). Harman’s single-factor test was conducted through explanatory factor analysis to examine the common method variance issue (e.g., Andersson and Bateman, 1997; Krishnan et al., 2006). All variables were examined through an exploratory factor analysis using the unrotated principal axis factoring procedure. If a substantial amount of common method variance is present, a single factor will emerge from the factor analysis, or one general factor will account for most of the covariance among variables. Results showed that six rotated factors emerged with an Eigenvalue greater than 1. The unrotated factors accounted for 67.82% of variance, and the first component only accounted for 26.60% of the total variance. Therefore, we believe that the common method variance was not of great concern, and unlikely to significantly confound the interpretation of results.

### Comparison Between the United States and Chinese Samples

We compared the scores of the major variables between the US and Chinese samples using multivariate analysis of variance (MANOVA). The results showed that the main effect was significant (*F* = 21437.26, *df* = 7, 2225, $\eta_{p}^{2}$ = 0.41, *p* < 0.01). Thereafter, a series of *T*-tests were performed to identify the group differences on each variable. Results indicated that compared with American participants, Chinese participants reported higher job demand (*M* = 2.90 and 2.47, *t* = 16.90, *p* < 0.01), job autonomy (*M* = 3.06 and 2.80, *t* = 8.42, *p* < 0.01), workaholism (*M* = 22.99 and 16.39, *t* = -27.13, *p* < 0.01), professional inefficacy (*M* = 10.31 and 8.44, *t* = -6.29, *p* < 0.01), but lower emotional exhaustion (*M* = 12.10 and 13.83, *t* = 4.85, *p* < 0.01). Table 2 presents the details of the comparison results.

**Table 2:** Comparison between China and the United States.

	United States		China		*t*
	*M*	*SD*	*M*	*SD*	
Job demands	2.47	0.58	2.90	0.61	16.90**
Job autonomy	2.79	0.75	3.07	0.79	8.42
Workaholism	2.34	0.79	3.28	0.84	-27.13**
Emotional exhaustion	2.77	1.69	2.42	1.67	4.85**
Depersonalization	2.66	1.69	2.72	1.44	-0.81
Personal accomplishment	1.41	1.06	1.72	1.27	-6.29**

^∗∗^*p < 0.01*.

### Correlation Analyses

Correlation analyses were conducted with participants from the three industries. Tables 3–5 presents the details of the correlation findings. For American participants in the natural resources and mining/construction industry, workaholism was positively related to both job demand (*r* = 0.48, *p* < 0.01), emotional exhaustion (*r* = 0.62, *p* < 0.01), and depersonalization (*r* = 0.43, *p* < 0.01). For Chinese respondents in the same industry, workaholism was also positively related to emotional exhaustion (*r* = 0.47, *p* < 0.01) and depersonalization (*r* = 0.35, *p* = 0.05).

**Table 3:** Correlation table – natural resources, mining and construction industry.

	1	2	3	4	5	6
(1) Job demand	–	0.29*	0.48**	0.28*	0.06	-0.41**
(2) Job autonomy	0.21	–	0.23	0.00	0.04	-0.15
(3) Workaholism	0.18	0.08	–	0.62**	0.43**	-0.05
(4) Emotional exhaustion	0.23	0.21	0.47**	–	0.71**	-0.13
(5) Depersonalization	0.11	-0.02	0.35*	0.77**	–	-0.10
(6) Personal accomplishment	-0.13	-0.12	0.09	0.00	0.22	–

*Data from the American sample are above the main diagonal and data from the Chinese sample are below the main diagonal. ^∗∗^p < 0.01, ^∗^p < 0.05.*

**Table 4:** Correlation table – manufacturing industry.

	1	2	3	4	5	6
(1) Job demand	–	0.19**	0.42**	0.33**	0.18**	-0.14*
(2) Job autonomy	0.10	–	0.10	-0.12*	-0.07	-0.28**
(3) Workaholism	0.39**	0.05	–	0.51**	0.42**	-0.11
(4) Emotional exhaustion	0.13	-0.34**	0.40**	–	0.68**	0.02
(5) Depersonalization	-0.08	-0.39**	0.14	0.71**	–	-0.08
(6) Personal accomplishment	-0.16	-0.45**	-0.13	0.24*	0.44**	–

*Data from the American sample are above the main diagonal and data from the Chinese sample are below the main diagonal. ^∗∗^p < 0.01, ^∗^p < .05.*

**Table 5:** Correlation table - service industry.

	1	2	3	4	5	6
(1) Job demand	–	0.18**	0.46**	0.36**	0.26**	-0.32**
(2) Job autonomy	0.00	–	0.13**	-0.10*	-0.12**	-0.26**
(3) Workaholism	0.43**	0.07*	–	0.47**	0.33**	-0.17**
(4) Emotional exhaustion	0.36**	-0.24**	0.37**	–	0.71**	-0.09*
(5) Depersonalization	0.12**	-0.23**	0.20**	0.69**	–	-0.11**
(6) Personal accomplishment	-0.10**	-0.23**	0.03	0.17**	0.39**	–

*Data from the American sample are above the main diagonal and data from the Chinese sample are below the main diagonal. ^∗∗^p < 0.01, ^∗^p < 0.05.*

In the manufacturing industry, American participants workaholism was positively related to job demand (*r* = 0.42, *p* < 0.01), emotional exhaustion (*r* = 0.51, *p* < 0.01) and depersonalization (*r* = 0.42, *p* < 0.01) among the American work sample. For the Chinese work sample, workaholism was positively related to job demand (*r* = 0.39, *p* < 0.01) and emotional exhaustion (*r* = 0.40, *p* < 0.01).

Finally, in the service industry, workaholism was positively related to job demand (*r* = 0.46, *p* < 0.01), job autonomy (*r* = 0.13, *p* < 0.01), emotional exhaustion (*r* = 0.47, *p* < 0.01), depersonalization (*r* = 0.33, *p* < 0.01), and negatively related to sense of personal accomplishment (*r* = -0.17, *p* < 0.01) among American participants. For the Chinese sample, workaholism was positively related to job demand (*r* = 0.43, *p* < 0.01), autonomy (*r* = 0.07, *p* = 0.05), emotional exhaustion (*r* = 0.37, *p* < 0.01), and depersonalization (*r* = 0.20, *p* < 0.01).

### Moderated Regression Analysis

We computed a series of moderated regression analyses to examine whether the effect of workaholism on job burnout differed across China and the United States. In each regression model, age, gender and country (with dummy code 0 = China, 1 = United States) were entered in the first step. In the second step, job demand, job autonomy and workaholism were entered. In the final step, the interaction term of country × workaholism was entered. Similar statistical procedure for using country as a moderator has been performed in earlier studies (e.g., Greenberger et al., 2000; Masuda et al., 2012). Following the recommendation by Frazier et al. (2004), workaholism was first centered before creating the interaction term. Burnout facets (i.e., emotional exhaustion, depersonalization, and personal accomplishment) were entered as the dependent variable of the regression models.

Tables 6–8 present the details of the moderated regression results. Across these regression models, workaholism was consistently related to emotional exhaustion and depersonalization across the three industry samples. In particular, workaholism was positively related to emotional exhaustion among participants from the natural resources, mining and construction industry (β = 76, *t* = 5.05, *p* < 0.01), manufacturing industry (β = 0.47, *t* = 7.41, *p* < 0.01), and the service industry (β = 40, *t* = 10.43, *p* < 0.01). Similarly, workaholism was also positively related to depersonalization among participants in natural resources, mining and construction industry (β = 41, *t* = 2.57, *p* < 0.01), manufacturing industry (β = 0.47, *t* = 7.41, *p* < 0.01), and the service industry (β = 0.30, *t* = 7.14, *p* < 0.05). Across these regression models, workaholism was not related to sense of personal accomplishment.

**Table 6:** Moderated regression analysis for natural resources, mining, and construction industry.

	Emotional exhaustion				Depersonalization				Personal accomplishment			
	*B*	*t*	*r*^2^	*F*	β	*T*	*r*^2^	*F*	β	*T*	*r*^2^	*F*
			0.03	0.92			0.14	4.57**			0.11	3.20*
Age	-0.13	-1.15			-0.20	-1.92			-0.25*	-2.42		
Gender	-0.09	-0.87			-0.26*	-2.55			-0.04	-0.40		
Country	0.09	0.81			0.19	1.84			0.19	1.79		
			0.36	7.43**			0.26	4.53**			0.23	3.97**
Age	-0.11	-1.18			-0.19	-1.94			-0.27**	-2.69		
Gender	0.06	0.60			-0.17	-1.63			-0.02	-0.16		
Country	-0.36	-3.16**			-0.06	-0.45			0.26*	2.05		
Job demand	0.05	0.43			-0.08	-0.69			-0.42**	-3.42		
Job autonomy	-0.04	-0.40			-0.03	-0.33			-0.04	-0.33		
Workaholism	0.73	5.68**			0.47**	3.40			0.17	1.23		
			0.36	6.32**			0.26	3.91**			0.24	3.50**
Age	-0.10	-1.03			-0.21	-2.03			-0.29	-2.82		
Gender	0.06	0.59			-0.17	-1.62			-0.02	-0.14		
Country	-0.35	-2.78**			-0.09	-0.65			0.22	1.59		
Job Demand	0.04	0.39			-0.08	-0.63			-0.41**	-3.31		
Job Autonomy	-0.04	-0.42			-0.03	-0.28			-0.03	-0.27		
Workaholism	0.76	5.05**			0.41**	2.57			0.10	0.60		
Country × Workaholism	-0.05	-0.39			0.10	0.65			0.13	0.87		

*^∗∗^p < 0.01, ^∗^p < 0.05.*

**Table 7:** Moderated regression analysis for manufacturing industry.

	Emotional exhaustion				Depersonalization				Personal accomplishment			
	*B*	*t*	*r*^2^	*F*	β	*T*	*r*^2^	*F*	β	*T*	*r*^2^	*F*
			0.01	1.70			0.00	0.42			0.02	2.43
Age	-0.08	-1.59			-0.05	-0.89			-0.11*	-2.20		
Gender	0.00	0.00			0.03	0.66			-0.01	-0.12		
Country	-0.07	-1.44			-0.02	-0.33			0.08	1.53		
			0.30	28.55**			0.15	12.00**			0.14	10.52**
Age	-0.01	-0.29			0.00	0.03			-0.14*	-2.91		
Gender	-0.02	-0.50			0.03	0.52			-0.03	-0.59		
Country	-0.29**	-6.11			-0.16**	-3.03			0.19**	3.60		
Job demand	0.14**	2.87			-0.01	-0.25			-0.07	-1.24		
Job autonomy	-0.24**	-5.46			-0.19**	-3.96			-0.31**	-6.50		
Workaholism	0.50**	9.58			0.40**	7.09			-0.09	-1.54		
			0.30	24.77**			0.16	11.17**			0.14	9.00**
Age	-0.01	-0.27			0.00	0.05			-0.14*	-2.91		
Gender	-0.03	-0.57			0.02	0.39			-0.03	-0.60		
Country	-0.29**	-5.88			-0.14**	-2.70			0.19*	3.59		
Job demand	0.14**	2.87			-0.01	-0.26			-0.07	-1.24		
Job autonomy	-0.24**	-5.50			-0.19**	-4.04			-0.31**	-6.49		
Workaholism	0.53**	9.15			0.47**	7.41			-0.08	-1.27		
Country × Workaholism	-0.07	-1.32			-0.13*	-2.32			-0.01	-0.23		

*^∗∗^p < 0.01, ^∗^p < 0.05.*

**Table 8:** Moderated regression analysis for tertiary industry.

	Emotional exhaustion				Depersonalization				Personal accomplishment			
	*B*	*t*	*r*^2^	*F*	β	*T*	*r*^2^	*F*	β	*T*	*r*^2^	*F*
			0.01	7.72**			0.00	2.23			0.02	10.80**
Age	-0.04*	-1.65			-0.04	-1.63			0.02	0.83		
Gender	0.00	-0.04			0.05	1.93			0.02	0.81		
Country	-0.11**	-4.44			0.02	0.84			0.13**	5.56		
			0.27	106.83**			0.12	37.38**			0.11	34.43**
Age	-0.02	-0.85			-0.02	-0.97			0.03	1.31		
Gender	-0.01	-0.25			0.04	1.84			0.01	0.60		
Country	-0.32**	-13.35			-0.09**	-3.47			0.21**	7.95		
Job demand	0.25**	10.22			0.09**	3.31			-0.21**	-7.77		
Job autonomy	-0.24**	-11.26			-0.22**	-9.64			-0.23**	-10.00		
Workaholism	0.37**	14.01			0.26**	8.94			0.06*	2.18		
			0.27	91.86**			0.12	32.39**			0.12	32.26**
Age	-0.02	-0.87			-0.02	-0.98			0.03	1.36		
Gender	-0.01	-0.29			0.04	1.79			0.02	0.75		
Country	-0.32**	-13.42			-0.10**	-3.63			0.22**	8.43		
Job demand	0.25**	10.19			0.09**	3.29			-0.21**	-7.73		
Job autonomy	-0.24**	-11.30			-0.22**	-9.69			-0.23**	-9.91		
Workaholism	0.40**	10.43			0.30**	7.14			-0.07	-1.59		
Country × Workaholism	-0.05	-1.34			-0.06	-1.50			0.16**	4.16		

^∗∗^*p < 0.01, ^∗^*p* < 0.05*.

**FIGURE 1:**
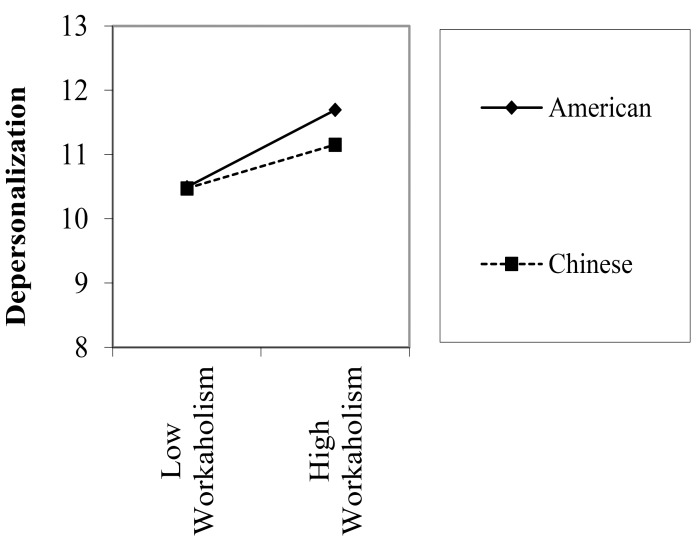
Moderated regression analysis predicting depersonalization among participants in manufacturing industry.

**FIGURE 2:**
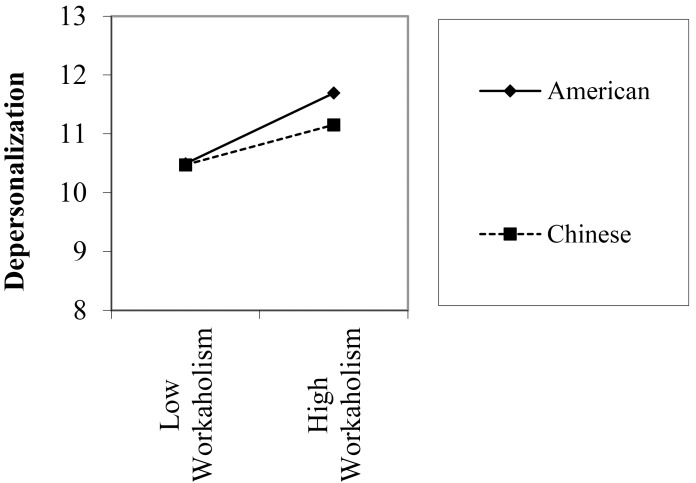
Moderated regression analysis predicting personal accomplishment among participants in service industry.

Finally, in relations to the moderating effect of country, only two out of nine interaction effects were significant. Figures 1, 2 graphically presented the significant moderation effects. The first set of significant moderating effect was found when country × workaholism in predicting depersonalization among manufacturing industry. In particular, depersonalization did not differ between American and Chinese participants when workaholism was low. However, American participants reported higher depersonalization than Chinese participants under high workaholism condition. Similarly, in predicting personal accomplishment among employees in service industry, sense of personal accomplishment did not differ between Chinese and American participants under low workaholism condition. However, in high workaholism condition, American participants reported lower sense of personal accomplishment when compared to the Chinese participants.

Taken together, workaholism was found to positively relate to emotional exhaustion and depersonalization across industries and countries. Therefore, H1 was supported. In H2, there was a lack of consistent moderating effect of country × workaholism in predicting burnout. So far, only two out of nine moderation effect was significant. Based on this result, H2 was only partially supported.

## Discussion

In this study, the goal was to examine the association between workaholism and job burnout, and whether this association differed across two culturally different contexts (i.e., China and the United States). In the regression analyses, workaholism was positively related to two burnout dimensions, namely emotional exhaustion and depersonalization in both China and the United States. We also found that the effect size of workaholism on the burnout dimensions varied: a medium effect between workaholism and emotional exhaustion was found, while the effect size was smaller with depersonalization. These findings are generally in line with previous studies that documented the significant association between the two constructs (e.g., Moyer et al., 2017; Schaufeli et al., 2009b). As suggested by the COR model (Hobfoll, 1989), workaholics tend to constantly engage in work, which will inevitable drain the employees’ valuable resources. At the same time, the high engagement in work also prohibit them to restore the resources through recovery. When the high investment of resource combines with the low replenishment of loses resources, this work pattern will contribute to higher job burnout.

To the best of our knowledge, our study is among the first to directly test the moderating effect of country on the relation between workaholism and job burnout. Although two significant moderating effects were found, results as a whole suggested that the relation between workaholism and job burnout did not vary remarkably across Chinese and American work samples. For the two significant moderating effect, we found that under low workaholism, both Chinese and American reported comparable level of depersonalization and sense of personal accomplishment. However, under high workaholism, American participants tend to report higher depersonalization and lower sense of personal accomplishment when compared to their Chinese counterparts. In individualistic cultures, such as in the United States, people are supposed to take care of themselves. When facing difficult situations, individuals are primarily coping with the work challenges by themselves. For collectivists (e.g., Chinese), they can easily mobilize social support from others to mitigate the negative consequence of workaholism. Based on the COR model, a plausible explanation is the availability of more social resources in the Chinese sample may help to buffer and alleviate part of the effect of workaholism, especially when workaholism is high. More research is warranted to examine whether the availability of resources differs across cultures which indirectly influence the effect of workaholism on burnout.

### Limitations

This study has several limitations and its results should be interpreted with caution. First, this research used a self-reported cross-sectional design, in which the participants were recruited during a single time point. Because of the nature of the data collection (cross-sectional data from a single source), common method variance is thus a potential statistical artifact which may bias the results. As discussed earlier, we had performed a Harman’s single factor test to evaluate the potential common method variance effect. Although the Harman’s single-factor test did not support the presence of common method variance factor that systematically inflate the correlations among observed factors, this procedure only provides a crude estimation of the common method variance, it did not directly remedy the statistical artifact which may bias the results (Podsakoff et al., 2003). As suggested by Podsakoff et al. (2003), in order to partial out the potential effect of common method variance, future can consider collecting additional factors, such as social desirability and to run different models (e.g., partial correlation, single-method-scale approach) or to obtain data from other sources (e.g., managers and coworkers) for external validation and running models to directly address the common method variance issues.

Second, we found that American and Chinese respondents reported different level of job burnout especially in the high workaholism condition. We speculated such difference originated from the availability of social support and their coping strategies. However, we did not directly assess these variables. In future studies, researchers could access these variables (e.g., perception of social support; stress-coping strategies or styles) and examine if these factors contribute to the observed differences between Chinese and American.

Third, we limited the scope of this study by analyzing the linkage between workaholism and burnout. Other important outcomes, such as the impact of work–family interface (e.g., work family interference), have not been addressed. The meaning and importance attached to family are considerably different when comparing collectivistic cultures (e.g., Chinese) and individualistic cultures (e.g., American). The inclusion of the family variable may provide incremental validity to understand how workaholism relates to other work- and health-related outcomes.

Fourth, internal consistency of the job demand scale in the Chinese sample was lower than the 0.70 convention (Nunnally and Bernstein, 1994). We cross-checked with the original items and compared with the translated items but we did not find any major discrepancies. Thus, future research should consider the inclusion of different measures to tap on this construct and for further validation. It is particularly important because the scale adopted in this study captured the overall job demands, future research may use other measurements to identify the connection between workaholism and different forms of job demands. For example, the use of multi-dimensional job demand scale (e.g., Bakker et al., 2005) will allow researchers to understand different forms of job demands (e.g., physical job demands, emotional job demands) in relations to workaholism. Such information will be useful in designing and implementing strategies to mitigate the influence of workaholism.

Finally, we found that the average weekly work hour reported was around 43 h. This number is generally equivalent to the average work hour of Mexico, which was found that be the nation that works the longest hour (OECD, 2018). However, whether our participants could be categorized as true work addicted or workaholic is still debatable. Therefore, even though we found a positive correlation between workaholism and burnout, we could only draw a preliminary inference on potential association between them.

### Implications

The results of this study indicate that regardless of cultures, workaholism is detrimental to the well-being of employees. Therefore, individuals and organizations should find ways to change. From an individual perspective, coping style plays an important role between workaholism and ill-health (Shimazu et al., 2010). For example, Shimazu et al. (2010) suggested that when facing workaholism, individuals could adopt more active coping strategies over emotional discharge as the former was positively associated with job performance and negatively related to self-reported ill-health. Besides, individuals should also actively monitor their work schedule and simultaneously attempt to strike a balance between work and activities, thereby enabling them to recover from exhaustion and fatigue.

Organization and leaders also play a strong role on workaholism. Van Wijhe et al. (2011) suggested that the change in business nature and the emergence of globalization increase market competition. To improve market edge, organizations often reward employees who are willing to work hard for a career, thereby inevitably increasing the pressure on employees and heighten workaholism. The creation of an organizational culture and reward system can enable employees to easily understand the management’s focus of attention. Workaholism will be increased when a subordinate perceives that workaholic behaviors will be rewarded. However, workaholism will be inhibited when such excessive work behaviors are not encouraged. Thus, to reduce the negative impact of workaholism, leaders should monitor their own behaviors and not impinge the idea of gaining reward from excessive work.

Lastly, organizations could perform periodical assessments of the psychological functioning of employees, including their workaholism tendency and general psychological health (i.e., job burnout and depressive symptoms). These assessment exercises enable organizations to organize and implement early intervention measures to enhance the psychological health of the workforce. For example, organizations could refer employees to career counseling service to assist them to strive for a healthy balance between work and other life domains.

## Ethics Statement

The project involves human subjects. The research protocol was reviewed and endorsed by the National University of Singapore Institutional Review Board for Social, Behavioral and Educational Research.

## Author Contributions

FC was responsible for drafting the manuscript and running the analyses. FC, CT, ML, and JK contributed to the theoretical development of the paper.

## Conflict of Interest Statement

The authors declare that the research was conducted in the absence of any commercial or financial relationships that could be construed as a potential conflict of interest.

## Footnotes

1 https://www.mturk.com/

2 https://sg.m.aipsurveys.com/aboutus
